# Isatin improves oligoasthenospermia caused by busulfan by regulating GSH/GPX4 axis to inhibit ferroptosis

**DOI:** 10.3389/fphar.2024.1489956

**Published:** 2024-10-31

**Authors:** Chengniu Wang, Weizhen Wang, Jin Dong, Xiaoran Li, Taowen Ye, Fanshuo Zeng, Mingyu Jiang, Jianwu Shi, Xiaorong Wang, Lei Zhang

**Affiliations:** ^1^ Institute of Reproductive Medicine, Medical School, Nantong University, Nantong, Jiangsu, China; ^2^ Center for Reproductive Medicine, Affiliated Maternity and Child Healthcare Hospital of Nantong University, Nantong, Jiangsu, China; ^3^ Department of Pharmaceutical Botany, School of Pharmacy, Naval Medical University, Shanghai, China

**Keywords:** isatin, oligoasthenospermia, ferroptosis, spermatogenesis, GSH/GPX4 axis

## Abstract

**Introduction:**

Ferroptosis, induced by iron overload and an imbalance in redox homeostasis, promotes the generation of reactive oxygen species (ROS), leading to iron-dependent lipid peroxides (LPO) and oxidative stress. Lipid peroxidation induced by reactive oxygen species is essential for the progression of spermatogenesis. However, its imbalance can lead to reproductive system damage and oligoasthenospermia, a critical cause of oligoasthenospermia. Isatin (ISA) is a naturally occurring compound that is widely distributed in lobsters, crustaceans, shellfish and various plants. It exhibits significant antioxidant and anti-aging properties, suggesting its potential as a therapeutic agent for the treatment of oligoasthenospermia. This study aimed to investigate the effects and mechanisms of ISA on oligoasthenospermia and to elucidate the underlying molecular pathways.

**Methods:**

All mice were divided into normal group, model group and treatment group. Both model group and treatment group received a single intraperitoneal injection of 30 mg/kg BUS to create the model of oligoasthenospermia. After 2 weeks, the treatment group received different doses of 25, 50 and 100 mg/kg ISA by gavage for 28 days, and then mice were sacrificed and tested.

**Results:**

The results demonstrated that ISA effectively reversed busulfan-induced reproductive system damage in mice. This included the restoration of testicular histomorphology, improvement in sperm concentration and motility, regulation of serum sex hormone levels, and normalization of various oxidative indices in testicular tissue. Furthermore, ISA successfully reversed testicular ferroptosis by restraining the translocation of nuclear factor erythroid 2-related factor 2 (NRF2) into the nucleus and improved oligoasthenospermia through the glutathione (GSH)/glutathione peroxidase 4 (GPX4) axis.

**Discussion:**

ISA was found to effectively ameliorate oligoasthenospermia in mice, presenting a potential therapeutic option for patients with this condition.

## 1 Introduction

Currently, 13%–15% of childbearing couples worldwide suffer from infertility, with approximately half of the cases attributed to male factors (Zhao et al., 2022). Among male infertility causes, approximately 40% are categorized as idiopathic male infertility, meaning specific causes cannot be identified (Sengupta et al., 2022). These cases include asthenospermia, azoospermia, and oligoasthenospermia (Lu and Huang, 2012). Studies have demonstrated that the incidence of oligoasthenospermia, a common cause of male infertility, has gradually increased over the past 50 years (Yang et al., 2022). Increased work-related stress, environmental pollution and some chemotherapy, are contributing factors to the gradual decline in both the quantity and quality of male sperm (Rosa-Villagrán et al., 2021; Zhao Y. et al., 2020). Althoughsome drugs are employed for clinical practice, their efficacy remains limited (Yilmaz et al., 2018). Consequently, it is necessary to explore new drugs for the treatment of male oligospermia. Therefore, exploring noveltherapies for oligoasthenospermia is important.

Isatin (2,3-Indolinedione, ISA) is a naturally occurring compound found in mammalian body fluids and tissues, as well as in various marine organisms like lobsters, crustaceans, shellfish, and certain plants and microbial metabolites (Gil-Turnes et al., 1989; Hosoe et al., 1999; Glover et al., 1988; Medvedev et al., 1996). ISA is particularly abundant in lobsters, where it is crucial for their survival (Hou et al., 2008). ISA and its derivatives are significant in fields such as anti-tumor, antiviral, and neuroprotection (Laird et al., 2000; Sriram et al., 2004; Ding et al., 2005). Research indicates that ISA’s concentration in mammalian reproductive organs is notably higher compared to other tissues (Glover et al., 1991). Besides, ISA exhibits anti-inflammatory and antioxidant properties and can inhibit liver cancer by upregulating the Nrf2 signaling pathway (Tawfik et al., 2022). Additionally, research suggests that ISA can improve spermatogenic function in male mice with gossypol acetate-induced spermatogenesis impairment (Guo et al., 2018). This effect may be attributed to its antioxidant properties and potential anti-inflammatory properties. Based on this, we speculate that ISA represents a promising avenue for treating oligoasthenospermia.

Ferroptosis, induced by iron overload and an imbalance in redox homeostasis, promotes the generation of reactive oxygen species (ROS), leading to iron-dependent lipid peroxides (LPO) and oxidative stress (Camaschella, 2015). Oxidative stress caused by free radicals is a significant contributor to male oligoasthenospermia, which can inflict substantial damage on reproductive system cells and disrupt spermatogenesis (Asadi et al., 2017). Studies have shown that the morphological abnormalities observed in rat sperm due to deficiencies in magnesium and zinc are associated with increased iron content and the generation of oxygen free radicals (Merker et al., 1996a; Merker and Günther, 1997; Merker et al., 1996b). Hence, the maintenance of proper testicular cell function necessitates the establishment of a balance between the production of free radicals and their metabolism, testes cells and tissues suffer severe damage (Romeo et al., 2004).

Currently, recognized mechanisms of ferroptosis regulation include the GSH/GPX4 axis, the NAD(P)H-FSP1-CoQ10 system, and the GCH1-BH4-DHFR system (Zheng and Conrad, 2020). The GSH/GPX4 axis regulation of ferroptosis is divided into three parts: 1. Regulate glutamate-cystine reverse transport system (System Xc-) (Chen et al., 2021). 2. Regulate Fe^2+^ and polyunsaturated fatty acid (PUFA) transport (Liu et al., 2022), 3. Affect Nrf2-HMOX1 axis (Yang et al., 2024). Inhibition of the GSH/GPX4 axis can reduce GPX4 production or accelerate GPX4 consumption through the above three pathways, resulting in an increase in LPO and ferroptosis. Busulfan (BUS), a critical chemotherapeutic agent, can reduce the number and functionality of spermatogonia, potentially leading to temporary or permanent male sterility (Ganjalikhan Hakemi et al., 2019). It has been reported to induce ferroptosis in spermatogonia by inhibiting the expression of GPX4 (Zhao X. et al., 2020; Xu et al., 2024). This study established a mouse model of oligoasthenospermia using BUS and investigated the potential of ISA treatment as a prospective clinical drug.

## 2 Materials and methods

### 2.1 Chemicals and reagents

ISA were purchased from Absin (Shanghai, China). BUS and dimethyl sulfoxide (DMSO) were supplied by Sigma-Aldrich (Shanghai, China). Hematoxylin-Eosin (HE) staining kit was purchased from Biosharp (Anhui, China). T, LH, FSH, malondialdehyde (MDA), superoxide dismutase (SOD), and reactive oxygen species (ROS) enzyme-linked immunosorbent assay (ELISA) kits was acquired from Herbal Source Biotechnology (Nanjing, China). Total iron assay kits was purchased from Yuanye Bio-Technology (Shanghai, China). Glutathione ELISA kits was acquired from Meimian (Yancheng, China). AceQ^®^ Universal SYBR^®^ qPCR Master Mix was obtained from Vazyme Biotech (Nanjing, China). ACSL4, CD71/Transferrin, SLC7A11/xCT, Ferritin Heavy Chain antibody was purchased from ABclonal (Wuhan, China). HO-1/Heme Oxygenase 1, KEAP1 antibody was obtained from Wanleibio (Shenyang, China). GPX4 Polyclonal Antibody was purchased from Invitrogen (Carlsbad, CA). Gapdh Antibody and Anti-Rabbit IgG was obtained from Sigma-Aldrich (Shanghai, China). Actin-Tracker Green-488, Alexa Fluor 488-conjugated Goat Anti-Rabbit IgG (H + L) and Alexa Fluor 555-labeled donkey anti-mouse IgG (H + L) was purchased from Beyotime Biotechnology (Shanghai, China). Antifade mounting medium for fluorescence (with DAPI) was acquired from Biosharp (Anhui, China).

### 2.2 Animals

Male ICR mice (n = 75, 8 weeks old, weighing 32 ± 2 g) were obtained from the Experimental Animal Center of Nantong University (Nantong, China). The mice were housed in standard laboratory conditions, with a room temperature maintained at 22°C–24°C and a 12/12-h light/dark cycle. They were provided *ad libitum* access to food and water. Our study protocol was reviewed and approved by the Animal Experiment Ethics Committee of Nantong University. All experimental procedures were conducted in accordance with the guidelines provided by the Laboratory Animal Center of Nantong University, with approval ID: P20230213-005.

### 2.3 Experimental design

BUS was dissolved in a 5% aqueous solution of dimethyl sulfoxide (DMSO), while the ISA preparation was formulated using 1% sodium carboxymethylcellulose solutions. Fifty mice were randomly allocated into five groups: the normal group, BUS group, BUS + 25 mg/kg ISA group, BUS + 50 mg/kg ISA group, and BUS + 100 mg/kg ISA group, with 10 mice in each group. We selected 50 mg/kg ISA based on previous pilot experiments (Supplementary Figure S1). The group treated with BUS and 50 mg/kg of ISA was referred to as the ISA group, and this dose was used in subsequent studies. Except for the normal group, the mice received a single intraperitoneal injection of BUS at a dose of 30 mg/kg (Wang et al., 2010). After a 2-week interval, the mice were administered different concentrations of ISA. Following a 4-week treatment, The mice were anesthetized with 1% sodium pentobarbital by intraperitoneal injection at a dose of 50 mg/kg. Blood was collected by puncturing the retro-orbital sinus of the mice, and the serum was then centrifuged at 3,000 g for 10 min at 4°C. The serum samples were stored at −80°C for further analysis. Testes and epididymis samples were collected and weighed. The mice were euthanized via cervical dislocation. ISA had no obvious toxic and side effects on the internal organs of mice (Supplementary Figure S2).

### 2.4 Analysis of sperm

After mice euthanasia, expeditiously transfer one epididymal tail to a Tyrode’s solution preheated to 37°C. Thoroughly mince the epididymal tail and continue incubation at 37°C for 15 min, allowing sufficient time for sperm to emerge. The computer-assisted analysis system was used to detect sperm concentration and sperm motility. The Hamilton Thorne CEROS II system is designed for both human and animal applications.

### 2.5 Histology

The tissues were placed in paraformaldehyde after 12 h, followed by dehydration using a sucrose gradient solution of 10%, 20%, and 30%, with tissue settling as the criterion for each dehydration step (Wang et al., 2020). Frozen sections with a thickness of 10 μm were prepared using a cryostat and stored at −20°C for later use. Hematoxylin and eosin (Biosharp, Anhui, China) staining was conducted following the instructions, and observations were made under an inverted microscope.

### 2.6 Western blot

After homogenizing all tissues, RIPA lysis buffer was employed to lyse the samples at 4°C for 20 min. Subsequently, the lysates underwent centrifugation at 12,000 g for 20 min, and the resulting supernatant was collected as tissue protein. After adding an appropriate loading buffer, the protein samples were denatured by heating at 95°C for 10 min. Following electrophoresis and transfer, membranes were blocked with 5% skimmed milk at room temperature for 1 h. Next, the membranes were subjected to overnight incubation with the primary antibody at 4°C, followed by incubation with the secondary antibody at room temperature for 1 h the next day (Wang et al., 2024). Enhanced chemiluminescence reagent (BL523A, Biosharp, Anhui, China) was utilized for visualization. Primary antibodies used include: GPX4 (PA5-102521, Invitrogen, 1:1,000), KEAP1 (WL03285, Wanleibio, 1:1,500), TFR1 (A21622, ABclonal, 1:100), FTH1 (A19544, ABclonal, 1:100), NRF2 (12721S, Cell Signaling Technology, 1:500), SLC7A11 (A2413, ABclonal, 1:750), HMOX1 (WL02400, Wanleibio, 1:1,000), P53 (AG3444, Beyotime, 1:1,000) and GAPDH (G9545-200ul, Sigma, 1:7,500).

### 2.7 Immunofluorescence analysis

After removing the frozen sections from −20°C, they were air-dried at 37°C for half an hour to prevent detachment. Permeabilization was achieved with 0.1% Triton for 40 min. After a 1-h incubation at room temperature, Actin-Tracker Green-488 (C2201S, Beyotime, 1:100) was ready for antibody retrieval. For other fluorescence staining, after blocking with 1% donkey serum at room temperature for 1 h, the primary antibodies were appropriately diluted and incubated overnight at 4°C, followed by PBS washing the next day. Subsequently, the sections were incubated with the secondary antibodies at room temperature for 1 h using the appropriate dilution ratio. Finally, the sections were washed three times with PBS, each time for 5 min. Subsequently, the sections were mounted using a mounting medium containing DAPI (BL739B, Biosharp, Anhui, China) for nuclear counterstaining (Wang et al., 2024; Dong et al., 2024). Observations were made using a confocal fluorescence microscope (TCSSP8, Leica). The primary antibodies detected by Immunofluorescence were ZO-1 (21773-1-AP, Proteintech, 1:1,000), GPX4 (PA5-102521, Invitrogen, 1:200), TFR1 (A21622, ABclonal, 1:100), NRF2 (12721S, Cell Signaling Technology, 1:500) and the secondary antibodies used include Alexa Fluor 555-labeled IgG (A0453, Beyotime, 1:500) and Alexa Fluor 488-labeled IgG (A0423, Beyotime, 1:500).

### 2.8 Enzyme linked immunosorbent assay for T, LH, FSH, GSH and total iron

Remove the serum from the −80°C freezer and allow it to thaw gradually. Levels of testosterone (T) (cby24183, Herbal Source Biotechnology, Nanjing, China), follicle-stimulating hormone (FSH) (cby24155, Herbal Source Biotechnology, Nanjing, China), luteinizing hormone (LH) (cby24209, Herbal Source Biotechnology, Nanjing, China), glutathione (GSH) (MM-0661M2, Meimian, Yancheng, China) and Total iron concent (R22186, Yuanye Bio-Technology, Shanghai, China) were measured by enzyme linked immunosorbent assay kits according to the manufacturer’s instructions. The minimum detectable dose of T, FSH, LH and GSH typically less than 0.0375 nmol/L, 0.125 U/L, 17.5 pg/mL and 0.625 ng/mL, respectively. The sensitivites of Total iron is 0.625 ng/mL and the detection range of it is above 0.1 μg/mL and below 40 μg/mL. The intra- and inter-assay coefficient of variation were <10% for all assays.

### 2.9 Enzyme linked immunosorbent assay for MDA, ROS and SOD

Testicular tissue was removed from the −80°C refrigerator, weighed, and subsequently cut. Pre-frozen PBS (pH 7.4) was added at a ratio of 1:9, and the mixture was thoroughly homogenized on ice using a glass homogenizer. The resulting liquid was centrifuged at 5,000 g for 10 min at 4°C, and the supernatant was carefully collected. Levels of malondialdehyde (MDA) (cby23821, Herbal Source Biotechnology, Nanjing, China), ROS (cby18879, Herbal Source Biotechnology, Nanjing, China), superoxide dismutase (SOD) (cby23823, Herbal Source Biotechnology, Nanjing, China) were measured by enzyme linked immunosorbent assay kits according to the manufacturer’s instructions. The sensitivities of ROS, MDA and SOD assays were 5 IU/mL, 0.075 nmol/L and 0.78 IU/mL, respectively. The intra- and inter-assay coefficient of variation were less than 10%.

### 2.10 RNA sequencing and data analysis

Total RNA of testes was extracted with Trizol reagent (R411, Vazyme, Nanjing, China). RNA concentrations were ascertained via a NanoDrop spectrophotometer (Thermo Fisher Scientific), and integrity was confirmed using a 2,100 Bioanalyzer (Agilent Technologies, San Diego, CA, United States). The PCR products were used to generate cDNA library, and then the BGISEQ-500MGISEQ-2000 system of BGI Shenzhen Branch was used for sequencing. Quality control and cleaning of the original data were performed using Fastp STAR comparison with reference genome GRCm38. Gene counts were obtained through Feature counts and the cluster Profiler software package was used for gene ontology (GO) analysis of differentially expressed genes.

### 2.11 Molecular docking

The molecular docking of ISA with proteins preformed using AutoDockTools-1.5.7 (https://ccsb.scripps.edu/mgltools/downloads/). Molecular Operating Environment software (https://www.chemcomp.com/Products.htm) was used to determine interactions between ligand and target proteins. The crystal structure of Nrf2 (Protein Data Bank [PDB] entry 3WN7), required for the docking studies, was obtained from the PDB (http://www.rcsb.org/structure/3WN7). The online website - PubChem – offers the structure of ISA, while utilizing the UniProt database for gene correction and identification.

### 2.12 Quantitative real-time PCR

The expression of related genes was further analyzed using real-time fluorescence quantitative PCR. Trizol reagent (R411, Vazyme, Nanjing, China) has been used to extract the total RNA of the mice testes. After confirming RNA concentrations and integrity, cDNA was synthesized using an RNA reverse transcription kit, and its concentration was measured with a NanoDrop 2000 spectrophotometer. The β-actin gene served as the internal reference. Three PCR reactions were conducted using ChamQ Universal SYBR qPCR Master Mix (Q711, Vazyme, Nanjing, China) on the CFX Connect system (Bio-RAD, United States). The sequences of Real-Time quantitative PCR primers can be found in Supplementary Table S1. Data analysis was performed based on the fold changes in mRNA expression (Ct).

### 2.13 Statistical analysis

All data are presented as mean ± SEM and were analyzed using GraphPad Prism 8.0. Student’s t-tests were employed to assess differences between two experimental groups, while one-way analysis of variance was utilized for comparing multiple experimental groups. A significance level of *P* < 0.05 was considered statistically significant.

## 3 Results

### 3.1 ISA reversed the BUS-induced oligoasthenospermia

**FIGURE 1:**
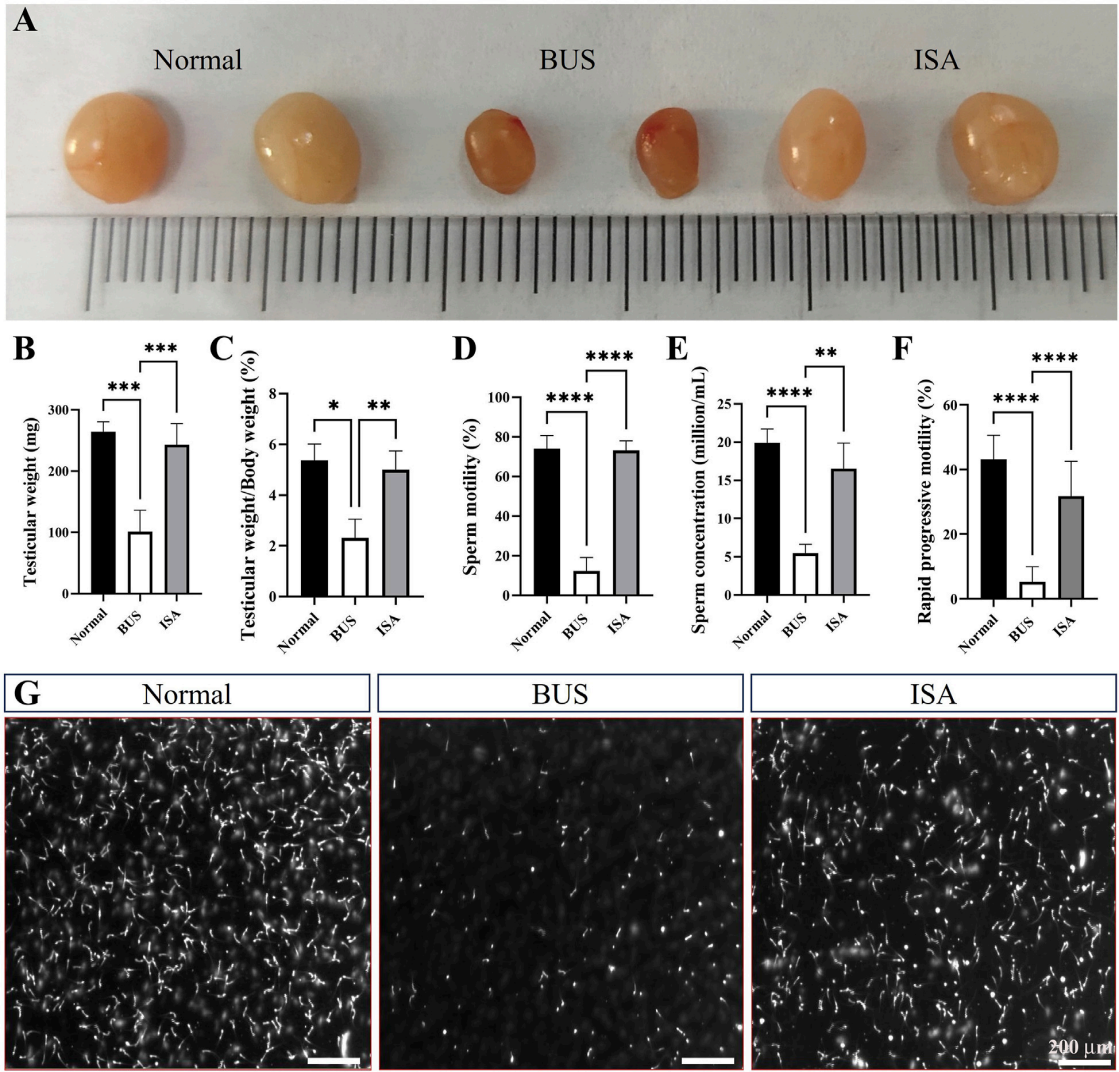
Effects on testicular organ index and sperm parameters. **(A)**: Morphology of the testes from 8-week-old mice after treatment with saline, BUS 30 mg/kg, BUS + ISA 50 mg/kg, **(B)**: Testicular weight, **(C)**: Ratio of testis weight to body weight, **(D)**: Sperm motility, **(E)**: Sperm concentration, **(F)**: Rapid progressive motility, **(G)**: Graph of sperm count, scale bar: 200 μm *P < 0.05, **P < 0.01, ***P < 0.001, ****P < 0.0001, n ≥ 8.

As illustrated in Figure 1, mice in the BUS group displayed testicular atrophy, reduced testicular weight, and a significantly decreased testis index compared to the normal group. Furthermore, there was a notable reduction in the proportion of rapidly progressive motility, sperm vitality and sperm concentration, indicating the successful establishment of the oligoasthenospermia model. Following ISA treatment, testicular weight increased in all three treatment groups (25 mg/kg, 50 mg/kg, and 100 mg/kg) (Supplementary Figure S1A). Additionally, sperm concentration, sperm vitality, and the proportion of rapidly progressive motility were all restored. However, there was no significant difference in sperm concentration between the BUS + 20 mg/kg ISA group and the BUS group (Supplementary Figures S1B–D). ISA administration at 50 and 100 mg/kg significantly reversed testicular injury in mice, therefore, we selected the 50 mg/kg dose of ISA based on prior pilot experiments and this dose was used in subsequent studies. The therapeutic efficacy of ISA in treating oligoasthenospermia was noteworthy.

### 3.2 ISA promoted the recovery of testis and epididymis histomorphology in oligoasthenospermia mice

**FIGURE 2:**
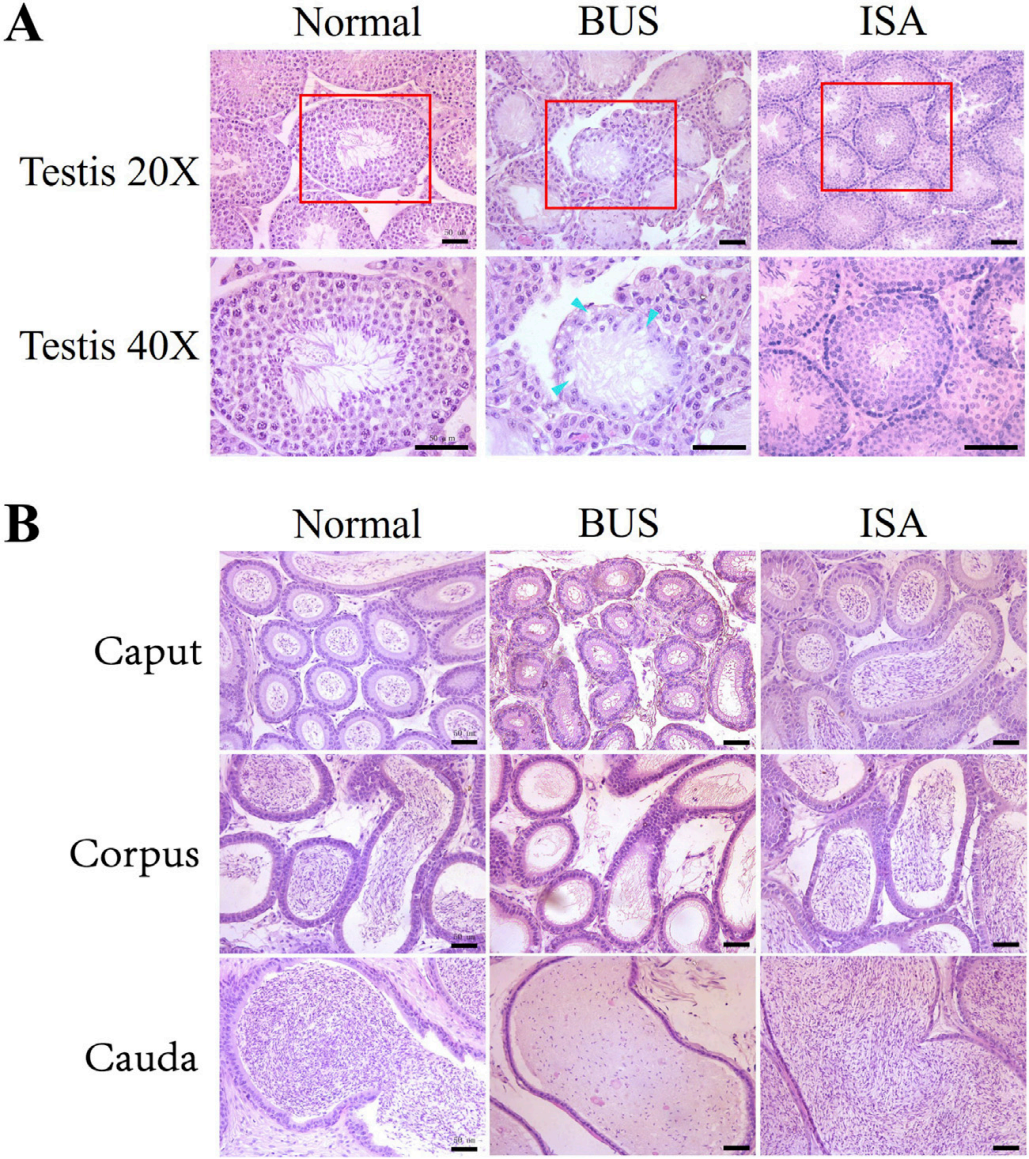
Histological examination of mice testes and epididymis. **(A)**: Morphology of testis. Blue arrow: vacuolization. **(B)**: Morphology of epididymis tail. Scale bars, 50 μm.

Our analysis revealed that BUS administration significantly depleted germ cells within the testes of mice and caused vacuolization, indicating a disrupted cellular arrangement and severe impairment of spermatogenesis and blood-testis barrier integrity, resulting in a Johnson score of 1–2. However, subsequent treatment with indigo carmine resulted in the reappearance of germ cells at various developmental stages from spermatogonium and spermatocyte to mature sperm, disappearance of vacuolization, and an increase in the diameter of seminiferous tubules, indicating the restoration of spermatogenesis, leading to a Johnson score of 7–8 (Figure 2A). The epididymis is the principal site for sperm maturation and vitality acquisition and serves as the primary sperm reservoir outside the seminal vesicle. As depicted in Figure 2B, BUS administration led to the absence of sperm in the caput, corpus, and cauda of the epididymis, along with detachment and shedding of the circular muscle surrounding the epididymis. Following ISA treatment, abundant sperm appeared in different segments of the epididymis, underscoring the significant therapeutic efficacy of ISA.

### 3.3 ISA remodels the cytoskeleton of seminiferous tubules and sperms

**FIGURE 3:**
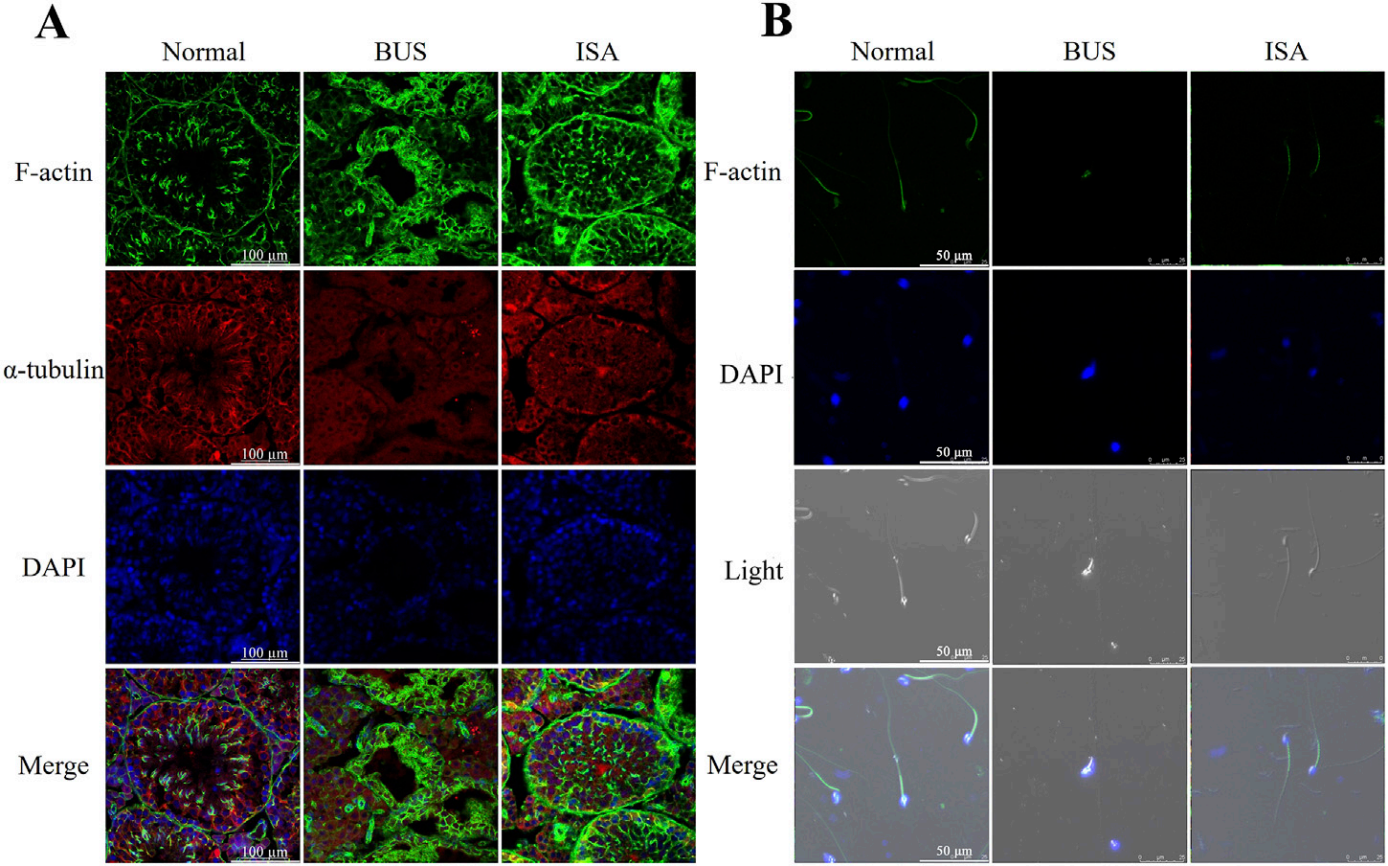
**(A)**: Changes of the cytoskeleton in different groups by immunofluorescence, scale bar: 100 μm. **(B)**: Changes of the cytoskeleton in sperm of different groups by immunofluorescence, scale bar: 50 μm.

To further validate the resuscitative effect of ISA on the testes of mice with oligoasthenospermia, we used immunofluorescence to observe the expression of microfilament and microtubule structures in seminiferous tubules and sperm (Figures 3A, B). In the BUS group, the overall organization of the testicular skeleton appeared disrupted, deviating from the typical “track-like” arrangement observed in healthy testes, suggesting exacerbated disruption of the cellular skeleton in mice with oligoasthenospermia and impeded reconstruction. However, following ISA treatment, a regular distribution of microfilaments and microtubules was evident, indicating a significant enhancement in the reconstruction of the testicular tubule cytoskeleton (Figure 3A). Furthermore, we found that morphology and cytoskeletal structure of sperm in BUS group were damaged, including head deformity or headlessness and detached necks. These issues were significantly improved after ISA treatment (Figure 3B).

### 3.4 ISA facilitates the restoration of compromised the integrity of blood-testis barrier

**FIGURE 4:**
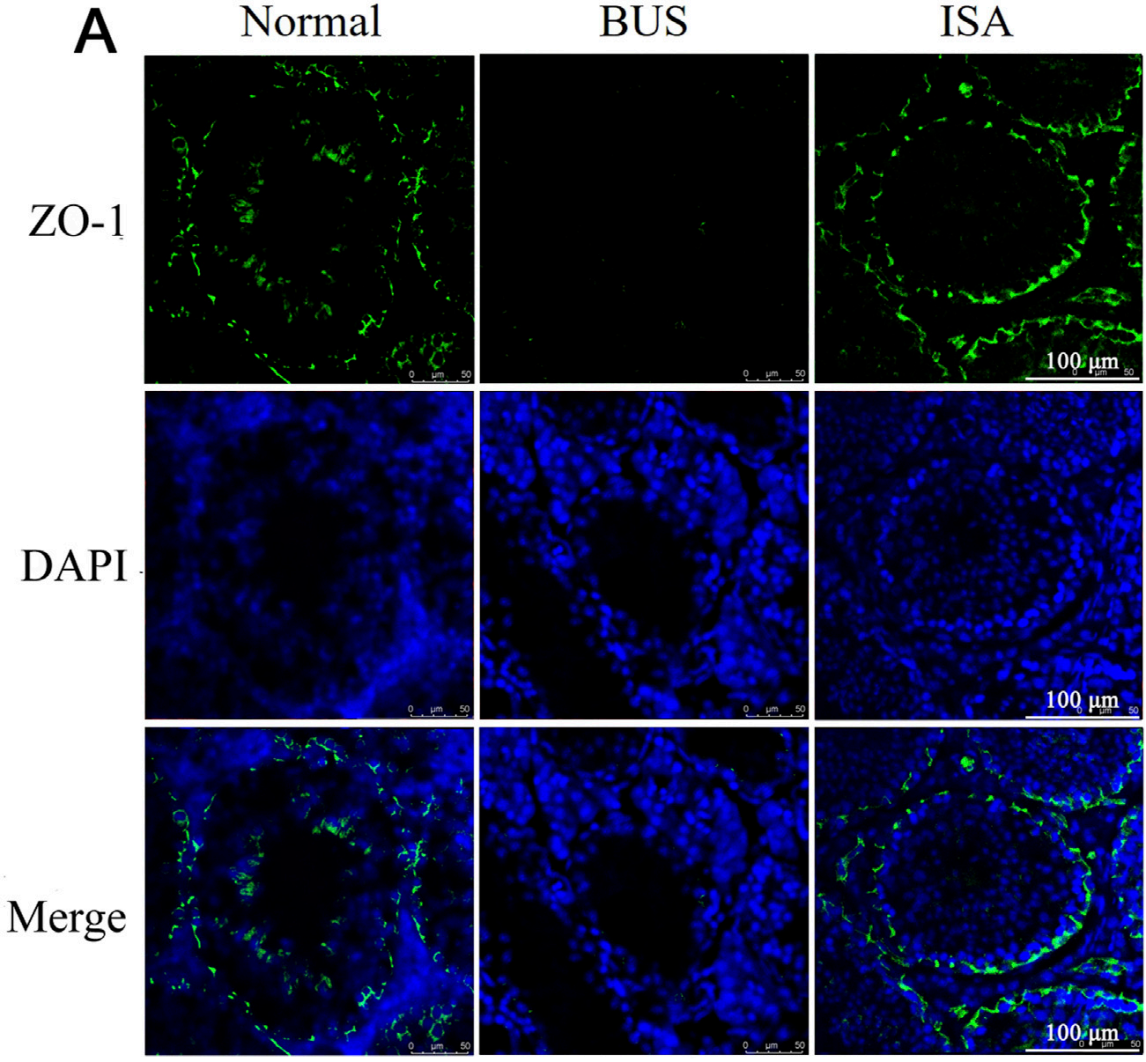
Immunofluorescent staining of ZO-1 protein in the testes of different groups. Green: ZO-1, Blue: DAPI, scale bar: 100 μm.

The Blood-Testis Barrier (BTB), a physical barrier between the blood vessels and seminiferous tubules in the testes, creates a stable microenvironment for spermatogenesis. To assess BTB integrity, we evaluated the expression of Zona Occludens 1 (ZO-1) protein in the testes. Immunofluorescence showed that ZO-1 protein expression was decreased and BTB integrity was destroyed in BUS group. Following ISA treatment, however, there was a restorative effect on the downregulation of the protein signal, thereby promoting the reconstitution of BTB integrity (Figure 4).

### 3.5 ISA raised testosterone levels in oligoasthenospermia mice

**FIGURE 5:**
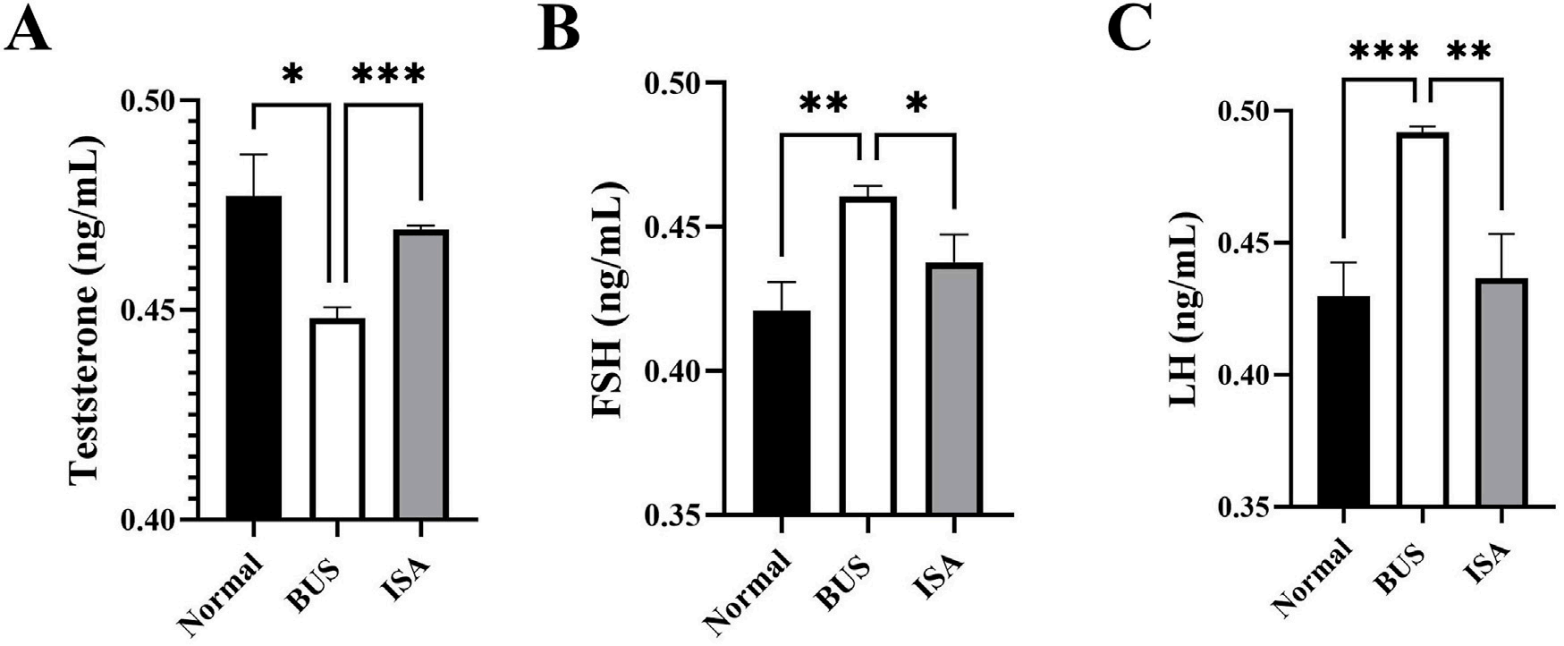
Effect of ISA on hormone levels. **(A)**: T concentration, **(B)**: FSH concentration. **(C)**: LH concentration. *P < 0.05, **P < 0.01, ***P < 0.001, n ≥ 3.

T, FSH and LH are key gonadal hormones that regulate the development and function of reproductive organs in living organisms. They also serve as crucial indicators of the reproductive system’s status. Consistent with previous studies, our comparison revealed that BUS-induced damage resulted in decreased serum testosterone levels in mice with oligoasthenospermia (Figure 5A). In response, negative feedback regulation in the hypothalamus and pituitary gland led to increased levels of FSH and LH (Figures 5B, C). However, following ISA treatment, we observed an increase in serum testosterone levels and a normalization of FSH and LH levels in the mice.

### 3.6 ISA improved oxidative indices in oligoasthenospermia mice

**FIGURE 6:**
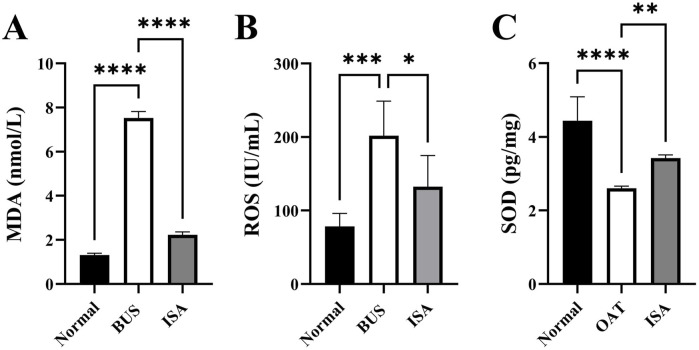
Effects of ISA on oxidative indices. **(A)**: MDA concentration, **(B)**: ROS concentration. **(C)**: SOD concentration. *P < 0.05, **P < 0.01, ***P < 0.001, ****P < 0.0001, n ≥ 3.

Oxidative stress is one of the important characteristics of oligoasthenospermia. As shown in Figure 6, we observed an increase in MDA and ROS levels, along with a decrease in SOD activity in testicular tissues of mice with oligoasthenospermia, indicating a decline in the antioxidant capacity and occurrence of oxidative stress to some extent. However, following ISA treatment, the levels of MDA and ROS in the testes of mice with oligoasthenospermia were reduced, while SOD activity increased. This suggests that ISA treatment suppressed the oxidative stress induced by BUS and restored antioxidant capacity in mice with oligoasthenospermia.

### 3.7 High-throughput sequencing and RT-qPCR validation

**FIGURE 7:**
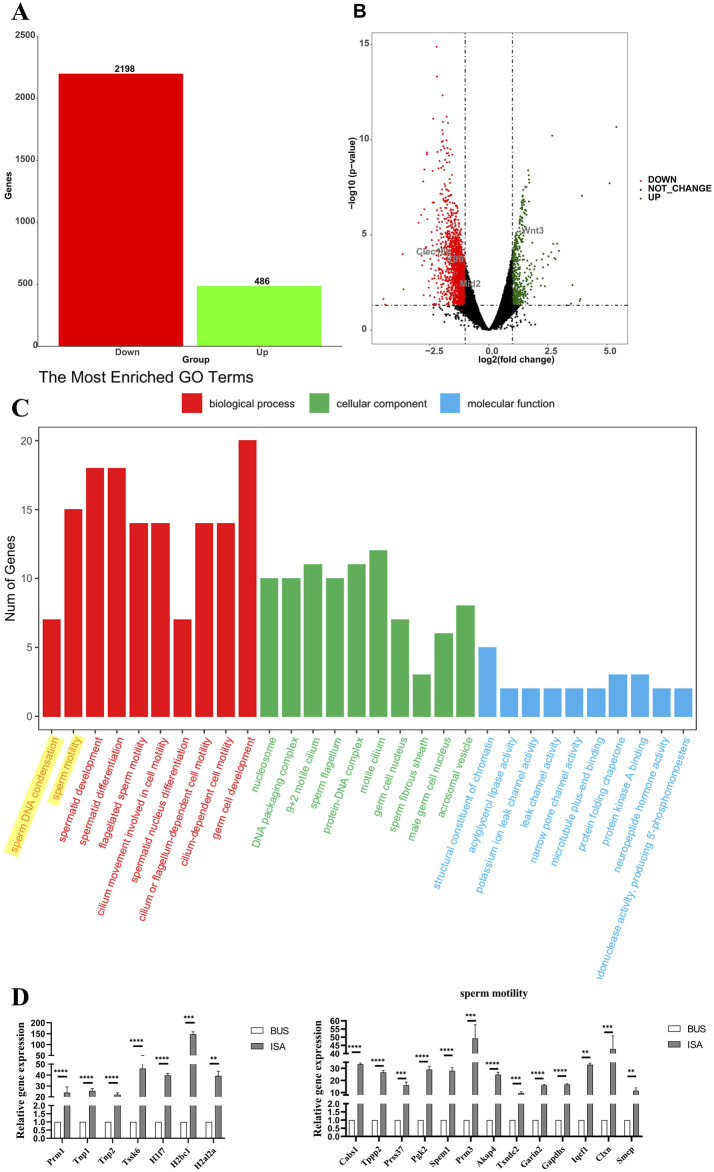
High-throughput sequencing. **(A)**: The number of differentially expressed genes **(B)**: Volcano map of differentially expressed genes between oligoasthenospermia and ISA treatments. **(C)**: GO enrichment analysis. **(D)** Verification of sequencing results by RT-qPCR. **P < 0.01, ***P < 0.001, ****P < 0.0001, n = 3.

After the completion of ISA treatment, high-throughput sequencing analysis revealed that ISA treatment resulted in the upregulation of 486 genes compared to the BUS group (Figures 7A, B). Subsequent analysis revealed that these genes were mainly involved in “sperm DNA condensation”, “sperm motility”, and “spermatid development and differentiation” (Figure 7C). We validated these sequencing results through RT-qPCR, which demonstrated that ISA treatment promoted spermatogenesis and sperm activation, thereby confirming the improvement of symptoms in mice with oligoasthenospermia. As shown in Figure 7D, based on the results of Gene Ontology (GO) analysis, we selected the most relevant pathways, “sperm DNA condensation,” and “sperm motility,” and validated the related genes identified by sequencing using RT-qPCR. The ISA group showed statistically significant upregulation compared to the BUS group, which was consistent with the sequencing results.

### 3.8 ISA inhibited ferroptosis by activating system Xc-

**FIGURE 8:**
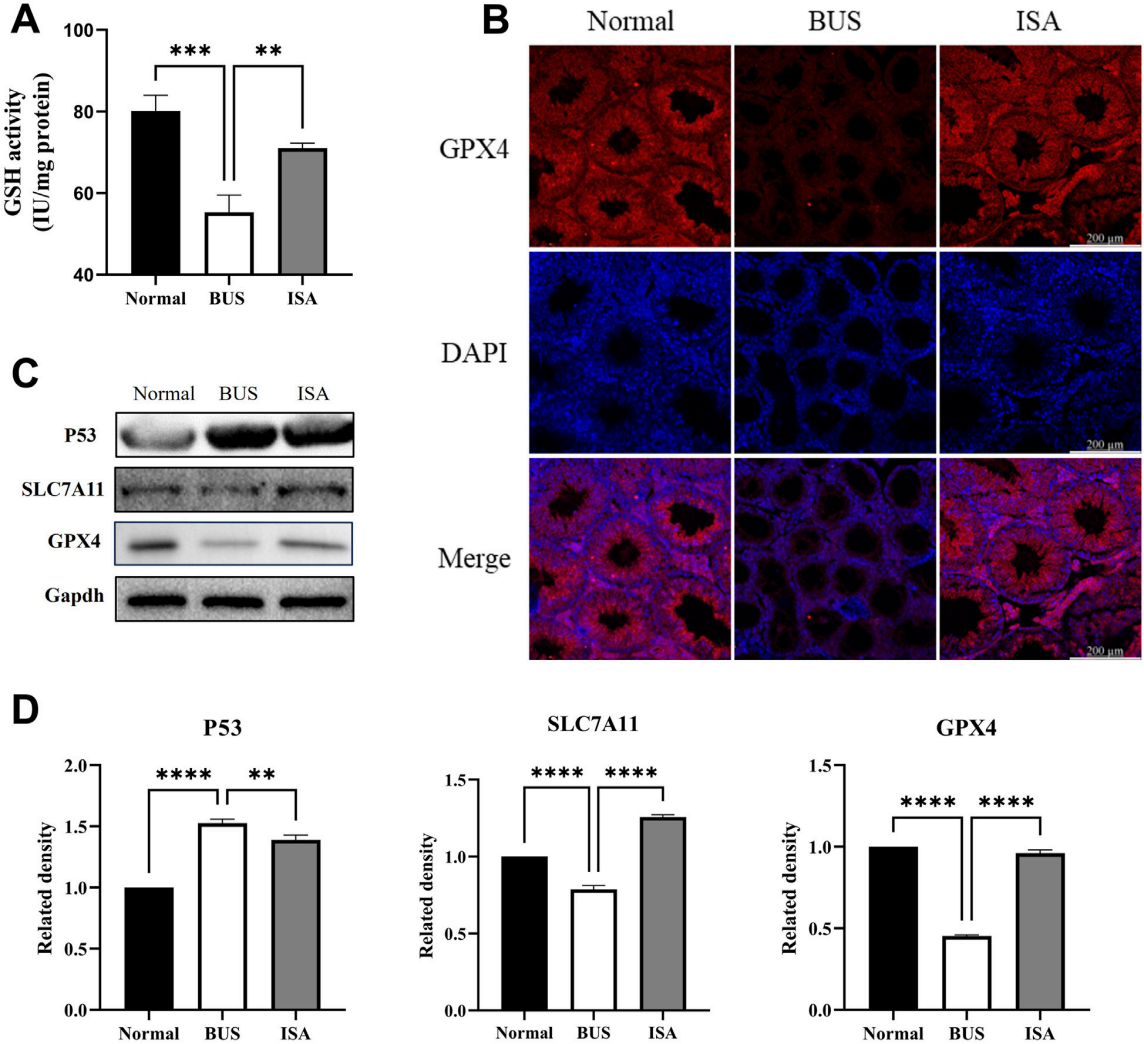
Effect of ISA on System Xc-. **(A)**: GSH activity. **(B)**: Immunofluorescence of GPX4. **(C, D)**: Western blot of System Xc-relative proteins. **P < 0.01, ****P < 0.0001, n = 3, scale bar: 200 μm.

We initially observed a decrease in glutathione (GSH) content and the expression of glutathione peroxidase 4 (GPX4) in busulfan-treated mice. This suggests that the GSH/GPX4 axis is inhibited in mice with oligoasthenospermia, leading to ferroptosis. It has been reported that p53 can inhibit SLC7A11 expression via transcription (Jiang et al., 2015) Further examination revealed that in busulfan-treated mice, p53 expression was upregulated, SLC7A11 expression was decreased, System Xc-was inhibited, and glutamate transport into cells was reduced. This resulted in diminished GSH synthesis, inhibition of GPX4 expression, and ferroptosis. Following ISA administration, P53 expression was inhibited, SLC7A11 was upregulated, System Xc-was promoted, GSH synthesis was enhanced, GPX4 expression was upregulated, and ferroptosis was inhibited. Immunofluorescence also showed low GPX4 expression in the busulfan group, whereas expression was restored after ISA treatment, further confirming these finding (Figure 8).

### 3.9 ISA inhibited ferroptosis by affecting Fe^2+^ and PUFA transport

**FIGURE 9:**
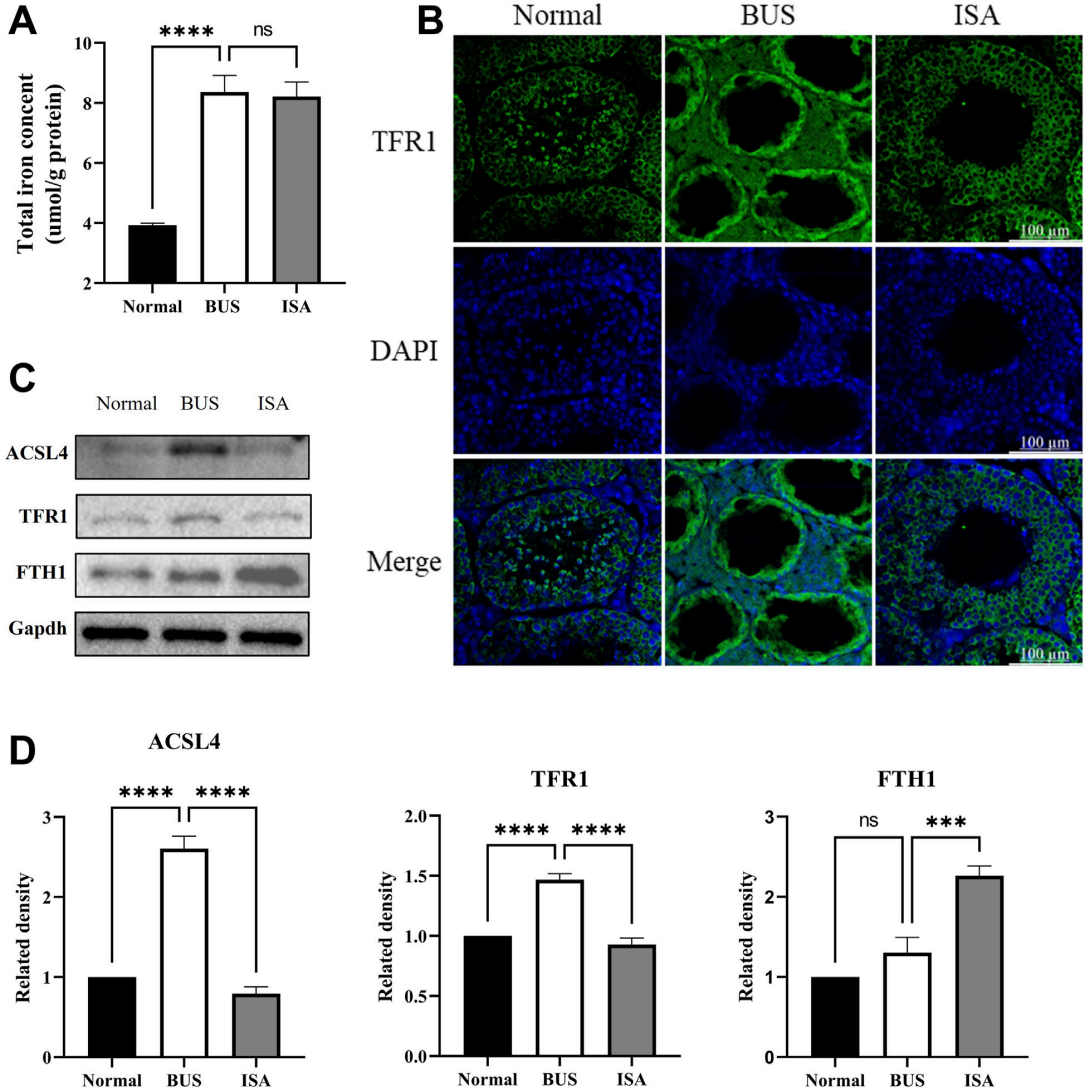
Effect of ISA on transport of Fe^2+^ and PUFA. **(A)**: Total iron concent. **(B)**: Immunofluorescence of TFR1. **(C, D)**: Western blot of Fe^2+^ and PUFA transport relative proteins. Ns: no significance, ***P < 0.001, ****P < 0.0001, n = 3, scale bar: 100 μm.

Compared to the control group, the testicular total iron content significantly increased in the busulfan group, accompanied by elevated expression of acyl-CoA synthetase long-chain family 4 (ACSL4) and transferrin receptor 1 (TFR1). While ferritin heavy chain 1 (FTH1) expression remained unchanged. Immunofluorescence reveals that TFR1 was expressed at all levels of spermatogenic cells in both the normal and ISA groups, with relatively low expression levels. However, in the BUS group, TFR1 was expressed in both seminiferous tubules and interstitial cells, with significantly higher expression. This suggests that BUS induces ferroptosis by facilitating the influx of polyunsaturated fatty acids (PUFA) and Fe^3+^ into cells, leading to ferroptosis (Figure 9). Following ISA administration, ACSL4 and TFR1 expression decreased, effectively inhibiting the transport of Fe^3+^ and PUFA. Concurrently, FTH1 expression increased, facilitating the binding of excess intracellular Fe^2+^ to inhibit ferroptosis. However, the total iron content did not exhibit a statistically significant decrease.

### 3.10 ISA inhibited ferroptosis by affecting Nrf2-HMOX1 pathway

**FIGURE 10:**
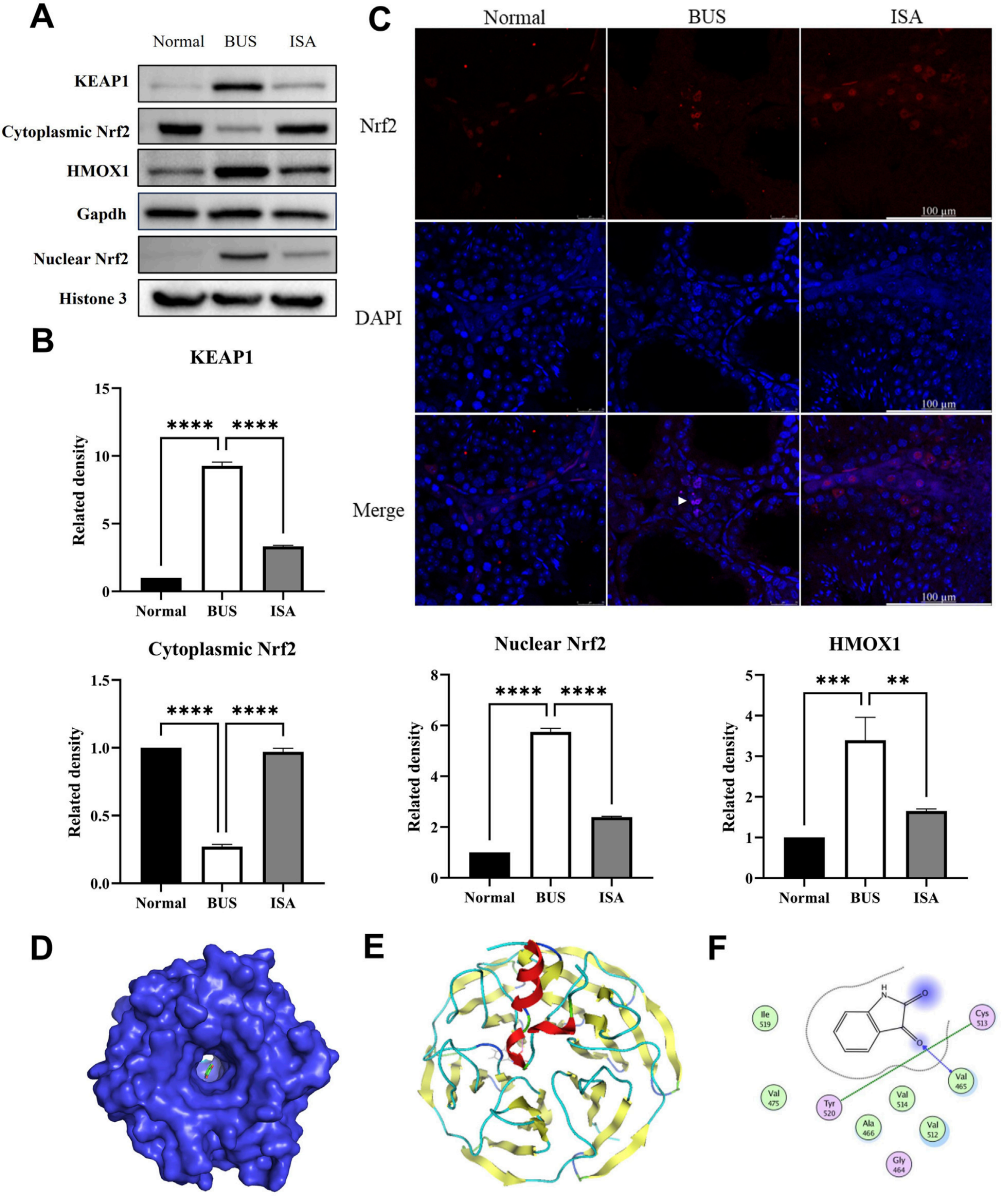
Effect of ISA on Nrf2-HMOX1 pathway. **(A, B)**: Western blot of Nrf2-HMOX1 pathway relative proteins. **(C)**: Immunofluorescence of Nrf2. **(D)**: Molecular docking of ISA with Nrf2. Grid score is −8.6349. **(E)**: Interaction plot between ISA and Nrf2. **(F)**: Interactive visualization of ISA binding to Nrf2 Val465, Tyr520, and Cys513 sites via hydrogen bonding. **P < 0.01, ****P < 0.0001, n = 3, scale bar: 100 μm.

Western blot analysis demonstrates that, compared to the normal group, the BUS group shown a significant increase in KEAP1 expression, a marked reduction in cytoplasmic Nrf2 expression and an elevation in nuclear Nrf2 expression. This suggests that BUS prompt the translocation of Nrf2 into the nucleus to exert antioxidant effects (Figures 10A, B). Immunofluorescence analysis confirms that Nrf2 is expressed in the cytoplasm of interstitial and Sertoli cells in normal testes, whereas in the BUS group, Nrf2 is localized in the nuclei of interstitial cells, further corroborating the Western blot results (Figure 10C). Concurrently, excessive heme oxygenase activity led to a substantial release of ferrous ions, generating numerous free radicals and resulting in extensive accumulation of lipid peroxides (LPO), culminating in ferroptosis. However, this damage was attenuated after ISA treatment. Molecular docking results showed that the grid score between ISA and Nrf2 is −8.6349. There is a hydrogen bond binding site between ISA and Nrf2 at Val465 site, and inter-residue contacts between Tyr520 site and cysteine Cys513 site in Nrf2 molecule occur (Figures 10D–F).

## 4 Discussion

In recent years, more and more couples have been troubled by infertility problems, and fertility problems caused by male infertility are becoming more and more common. Consequently, effective prevention and solutions for male infertility have garnered significant research attention. Oligoasthenospermia is one of the main factors affecting male fertility. Its etiology is complex, the mechanisms are not well understood, and there are no targeted treatments or drugs available in clinical practice. Therefore, this field presents substantial research opportunities.

Isatin, a naturally occurring compound with antioxidant properties, is widely found in both plants and animals. In the last century, studies have indicated that indigo is highly expressed in the reproductive system, however, no research has reported its therapeutic effects on male infertility (Glover et al., 1991). Our study demonstrated that treatment with 50 mg/kg of isatin effectively improves oligoasthenospermia in mice, evidenced by testicular size recovery, increased testicular weight, enhanced sperm motility and concentration, as well as improved tissue morphology and spermatogenesis (Figures 1–3). The blood-testis barrier (BTB) plays a critical role in maintaining spermatogenesis by creating a highly selective environment in the seminiferous tubules. It facilitates the selective passage of nutrients, ions, and hormones necessary for sperm maturation while blocking harmful substances, including immune cells and antibodies, from entering the adluminal compartment. Research indicates that impairments in the BTB can lead to the accumulation of immature germ cells, resulting in conditions such as oligospermia and oligoasthenospermia (Zhou and Wang, 2022). Our study demonstrates that ISA can restore the blood-testis barrier in the testes of oligoasthenospermia mice, potentially contributing to the recovery of testicular damage in these animals (Figure 4).

Furthermore, our study demonstrates that ISA treatment effectively relieves oxidative stress in oligoasthenospermia mice (Figure 6). Oligoasthenospermia is closely associated with oxidative stress (OS), which results from an excess of ROS. Elevated ROS levels can damage sperm cell membranes, proteins, and DNA, leading to sperm dysfunction and negatively impacting male fertility (Alvarez and Storey, 1995; Storey, 1997). Reductions in ROS and MDA levels, alongside increases in superoxide dismutase (SOD) levels *in vivo*, can effectively improve sperm parameters and alleviate sperm DNA fragmentation. A study conducted in 2011 demonstrated that supplementation with selenium and vitamin E significantly improved semen parameters, including sperm motility and morphology, in men with infertility (Alahmar, 2023). A more recent clinical trial conducted in 2023 investigated the effects of a multi-antioxidant formula containing L-carnitine, selenium, coenzyme Q10, and other micronutrients on men with oligoasthenoteratozoospermia. The treatment resulted in a significant reduction in sperm DNA fragmentation and improved clinical outcomes in assisted reproduction techniques, such as intracytoplasmic sperm injection, highlighting the importance of antioxidants in enhancing fertility (Lahimer et al., 2023; Rochdi et al., 2024). In our study, MDA content and ROS levels in the testes of mice in the ISA group were significantly reduced, while SOD content was significantly increased compared to mice in the BUS group (Figure 6). Malondialdehyde is a natural byproduct of LPO and is positively correlated with ferroptosis, serving as one of its biomarkers (Del Rio et al., 2005). These findings suggest that ISA significantly inhibited oxidative stress induced by BUS and facilitated the recovery of the antioxidant system in mice with oligoasthenospermia.

Increasing research indicates that male reproductive damage is closely associated with ferroptosis. Ferroptosis, a mode of cell death caused by excessive accumulation of iron-dependent lipid peroxides (LPO) (Tang et al., 2021), primarily results from three factors: an imbalance in System Xc-, disturbances in iron metabolism and abnormalities in the metabolism of polyunsaturated fatty acids (PUFAs). During development, male germ cells must undergo continuous division, with iron being essential for DNA synthesis (Tvrda et al., 2015). However, iron overload can lead to the expression of ferroptosis-related genes expression and the inactivation of associated enzymes, resulting in dysfunction of the male reproductive system (Kocpinar et al., 2020). In spermatogenesis, ferroptosis can lead to a significant reduction in the number of round spermatids, cells that lack cytoplasmic antioxidants and are highly sensitive to LPO, resulting in extensive cell apoptosis (Bromfield et al., 2019). Our research offers a potential pharmacological approach for the clinical treatment of oligoasthenospermia from the perspective of ferroptosis. Testicular iron overload, inactivation of the antioxidant enzyme GPX4, and lipid metabolism disorders contribute to the death of a significant number of cells associated with the male reproductive system, leading to reproductive disorders.

We have demonstrated that the System Xc-is activated by ISA, which inhibits ferroptosis in the mouse testes, thereby treating oligoasthenospermia (Figure 8). System Xc- is a transmembrane amino acid transport system that primarily imports L-cysteine into the cell while exporting glutamate to maintain cellular redox balance (Dixon et al., 2012). It is composed of two subunits: SLC7A11, which facilitates amino acid transport, and SLC3A2, an auxiliary subunit (Blahna and Hata, 2013). System Xc- plays a crucial role in cellular antioxidant defense by promoting the synthesis of glutathione (GSH) through the uptake of L-cysteine (Stockwell et al., 2017). In System Xc-, Glutathione is one of the most important antioxidants within the cell, and its presence is essential for inhibiting lipid peroxidation, particularly in the context of iron-dependent cell death known as ferroptosis (Dixon et al., 2012). Moreover, GSH is the main antioxidant in mammalian cells and the preferred substrate of GPX4. The consumption of GSH directly affects the activity and stability of GPX4, thus making cells more susceptible to ferroptosis. In our study, GSH expression was significantly reduced in the BUS group, indicating that GSH depletion occurred in oligospermic mice, increasing their susceptibility to ferroptosis. However, ISA treatment was able to reverse this effect (Figure 8). In System Xc-, SLC7A11 mediates cellular uptake of cysteine, a precursor for GSH synthesis, and inhibition of SLC7A11 protein expression disrupts intracellular redox balance, triggering ferroptosis (Shen et al., 2022). Besides, P53 antagonizes SLC7A11 (Jiang et al., 2015). Our experiments demonstrated that BUS administration resulted in increased expression of P53, decreased GSH, and suppression of SLC7A11 and GPX4, and that these changes were reversed by ISA administration, which reversed ferroptosis in oligoasthenospermia mice testis through activation of System Xc- (Figure 8).

Some studies have shown that iron overload can promote the production of free radicals, leading to LPO of cell membranes and ultimately to ferroptosis of cells (Chen et al., 2023). TFR1 is a functional protein that transports iron from the outside to the inside of the cell and is considered to be one of the specific ferroptosis markers (Feng et al., 2020). In this experiment, TFR1 was upregulated in testes of BUS group mice, which caused iron overload and ferroptosis. The increase of total iron level in testes of BUS group mice compared with normal group also verified this result (Figure 9). However, the expression of TFR1 was suppressed after ISA treatment, but the total iron level did not decrease significantly. Therefore, we think that the level of Fe^2+^ may be a more appropriate indicator of ferroptosis. ACSL4 is an enzyme involved in fatty acid metabolism, and upregulation of ACSL4 increases the content of PUFA in phospholipids, which are susceptible to oxidation and lead to ferroptosis (Gan, 2022). In this experiment, ACSL4 increased in BUS group compared with normal group, and decreased after ISA administration (Figure 9). ISA can inhibit ferroptosis by inhibiting the transport of Fe^2+^ and PUFA.

Nrf2, a key transcription factor involved in regulating antioxidant responses, can bind to the promoters of multiple antioxidant genes and antioxidant response elements (AREs), thereby inducing the expression of downstream genes (Metzendorf and Lind, 2010). KEAP1 normally binds to Nrf2 in the cytoplasm, but when mouse testes are stimulated by high ROS induced by BUS, Nrf2 dissociates from KEAP1 and is transferred into the nucleus for activation. This is consistent with our experimental results, and after ISA administration, ROS levels in tissues are reversed, and Nrf2 is re-associated with KEAP1 in the cytoplasm and highly expressed in the cytoplasm (Figure 10). HMOX1 is a rate-limiting enzyme in the heme catabolism of iron porphyrin compounds, which can decompose heme into carbon monoxide, biliverdin and ferrous ions (Fang et al., 2019). High expression of HMOX1 in BUS group leads to high concentration of ferrous ion-mediated ferroptosis, while the expression of HMOX1 is also significantly inhibited after ISA administration, i.e. ferroptosis is inhibited by regulating Nrf2-HMOX1 axis (Figure 10). However, whether ISA can regulate ferroptosis through NAD(P)H-FSP1-CoQ10 system or GCH1-BH4-DHFR system remains unclear and needs further study.

In addition to ferroptosis, apoptosis is also a significant contributor to male infertility. Several factors may contribute to male infertility, including oxidative stress, endocrine disorders, DNA damage, inflammatory responses, and mitochondrial dysfunction (Xu et al., 2023; Dai et al., 2018). Among these, studies have shown that oxidative stress, DNA damage and mitochondrial respiration defects may be the trigger of apoptosis, leading to oligoasthenospermia (Nakada et al., 2006; Zhao et al., 2021). Mitochondrial respiration defects caused by the accumulation of DeltamtDNA can arrest mouse spermatocytes at the zygotene stage and trigger apoptosis, which leads to oligoasthenospermia (Nakada et al., 2006). Thus, oxidative stress, a known trigger of ferroptosis, also contributes to apoptosis. Our study shows that ISA may inhibit the oxidative stress in oligoasthenospermia mice and primarily confirms that ISA may contribute to the recovery of oligoasthenospermia through ferroptosis but did not further study the mechanism of apoptosis. ISA has great potential to treat oligoasthenospermia by reversing apoptosis, which is a very promising research direction in the future.

## 5 Conclusion

**FIGURE 11:**
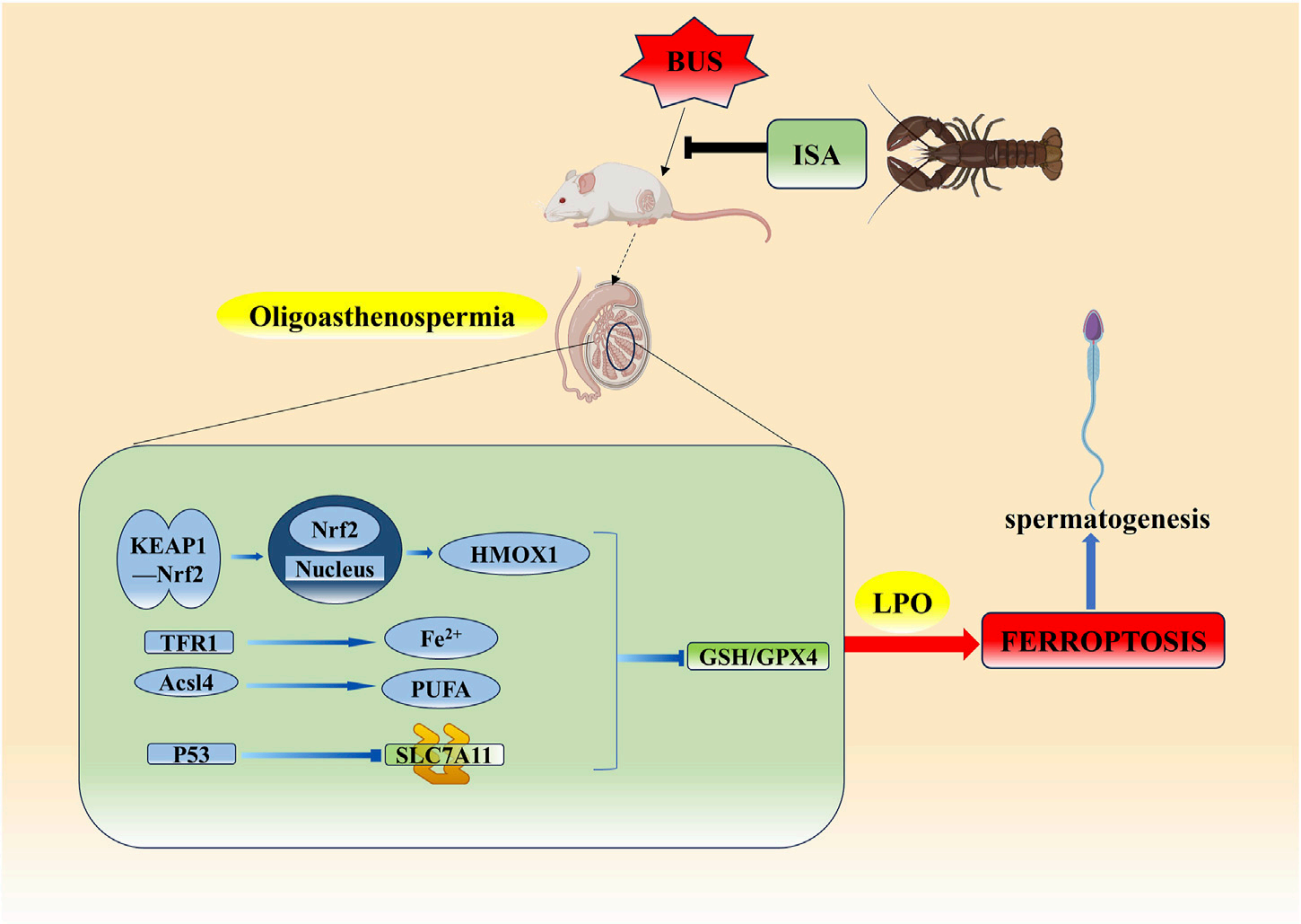
Possible mechanism of ISA on oligoasthenospermia mice induced by BUS.

In conclusion, ISA was found to be effective in ameliorating oligoasthenospermia induced by busulfan in mice. After ISA administration, sperm concentration and motility were significantly increased, serum sex hormone levels were regulated, and oxidative damage in tissues was significantly improved. It was clear that ISA could effectively inhibit ferroptosis and improve oligoasthenospermia through three different ways of GSH/GPX4 axis (Figure 11).

## Data Availability

The original contributions presented in the study are included in the article/Supplementary Material, further inquiries can be directed to the corresponding author/s.
