# Supplementary figures and images for: Isatin improves oligoasthenospermia caused by busulfan by regulating GSH/GPX4 axis to inhibit ferroptosis
Source: Front Pharmacol. 2024 Oct 31;15:1489956. doi: 10.3389/fphar.2024.1489956 (PMC11561459; doi:10.3389/fphar.2024.1489956)

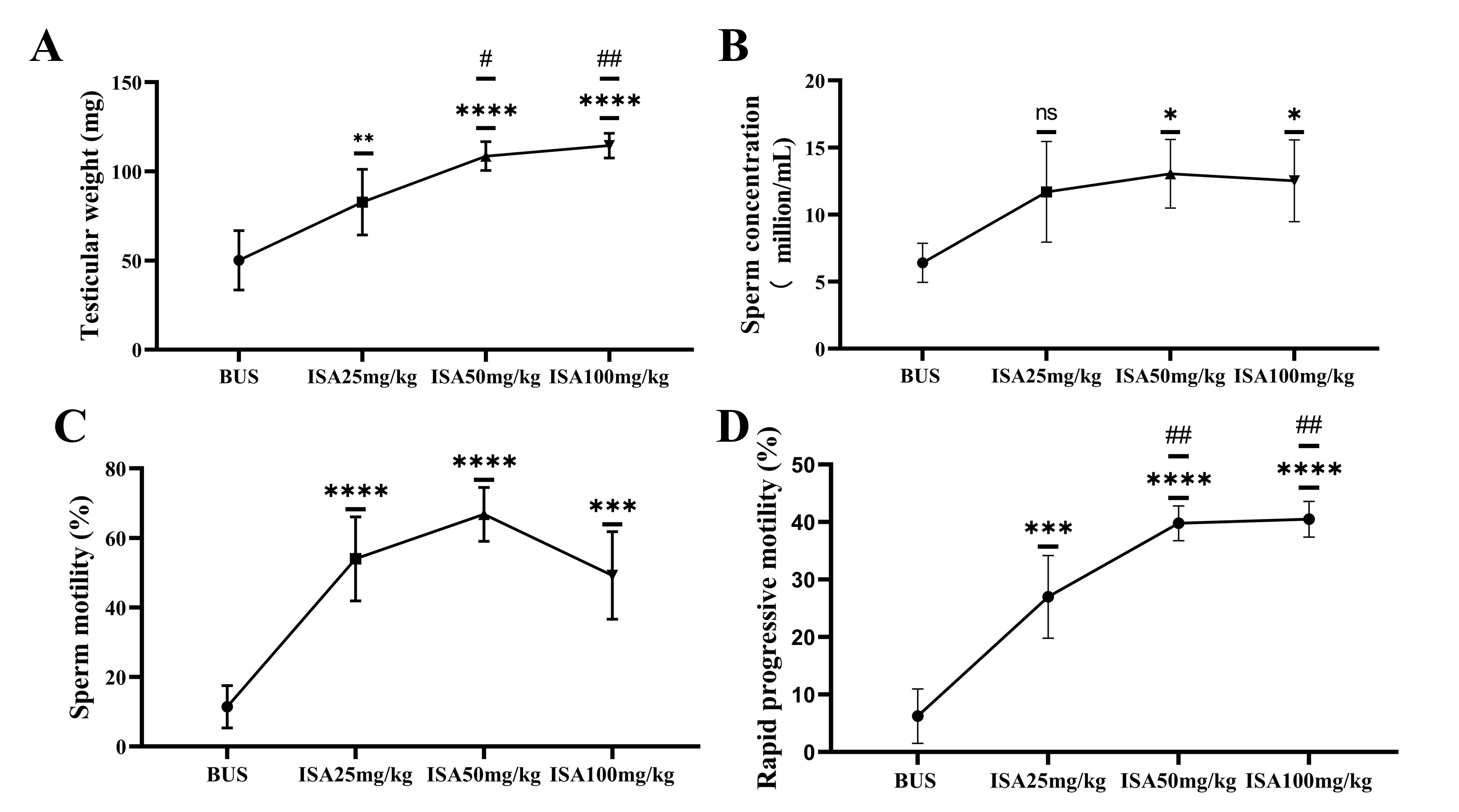

Supplement: Supplementary file 1 [file Image1.JPEG]

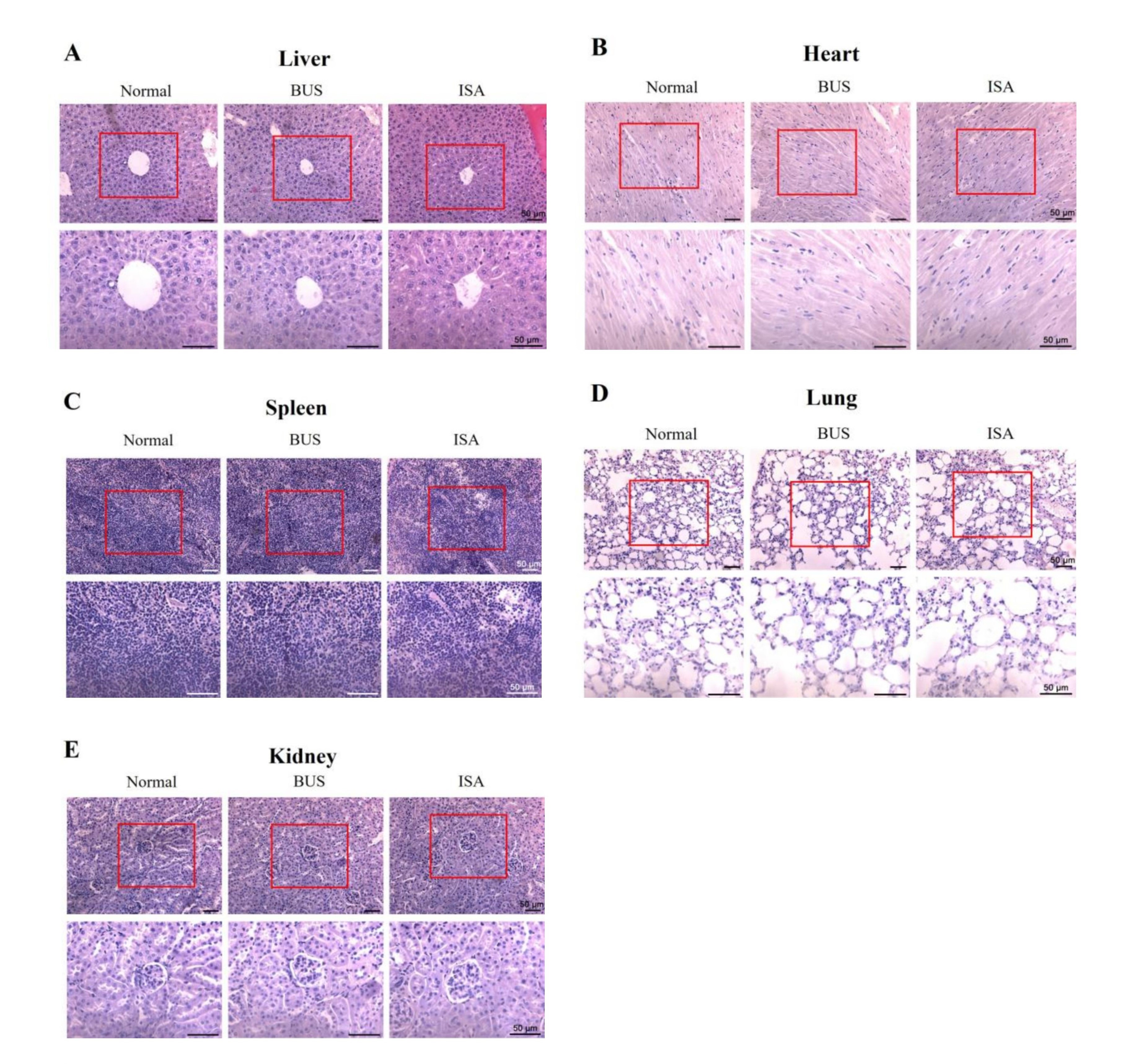

Supplement: Supplementary file 2 [file Image2.JPEG]
