# Supplementary material for: Isatin improves oligoasthenospermia caused by busulfan by regulating GSH/GPX4 axis to inhibit ferroptosis
Source: Front Pharmacol. 2024 Oct 31;15:1489956. doi: 10.3389/fphar.2024.1489956 (PMC11561459; doi:10.3389/fphar.2024.1489956)
Supplement: Supplementary file 4 [file DataSheet1.DOCX]

Supplementary Material


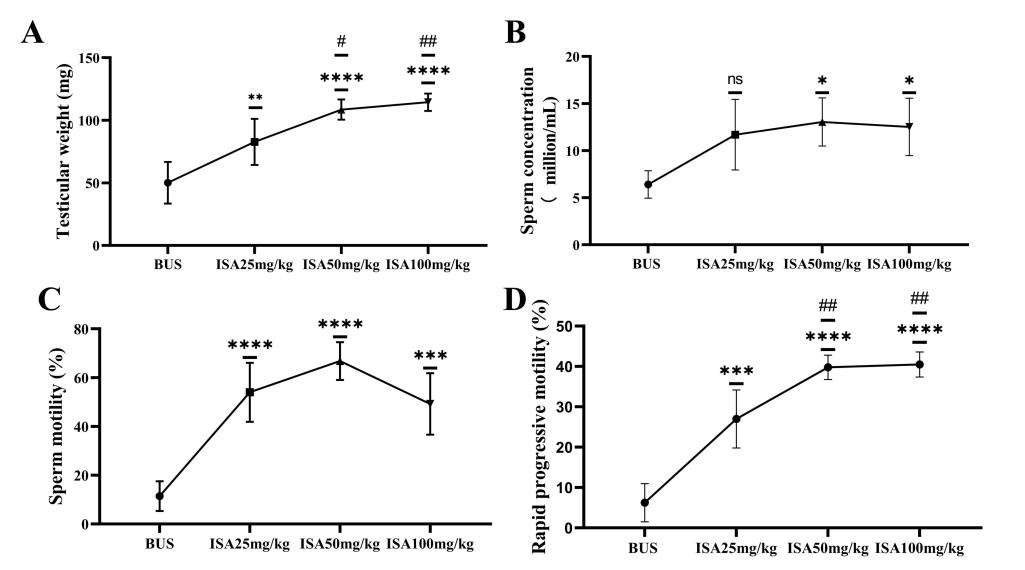


**Supplementary Figure 1.** Effects of different concentrations of ISA (Isatin) on testicular weight and sperm. (A): testicular weight, (B): sperm concentration, (C): sperm motility, (D): rapid progressive motility. *: vs. BUS group, #: vs. ISA 25mg/kg group. ns: no significance, *P<0.05, **P<0.01, ***P<0.001, ****P<0.0001, #P<0.05, ##P<0.01.


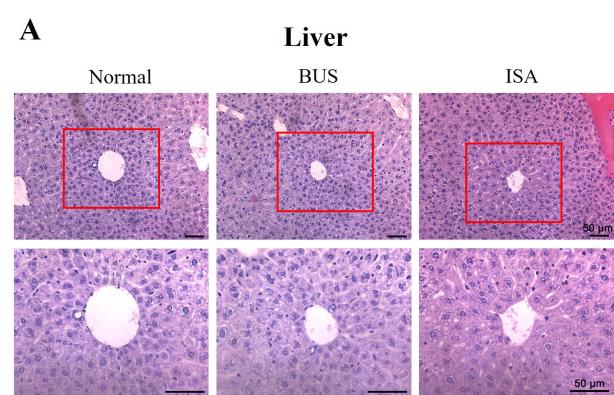

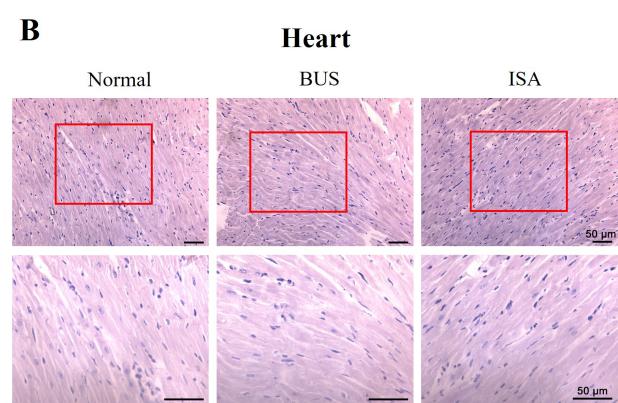

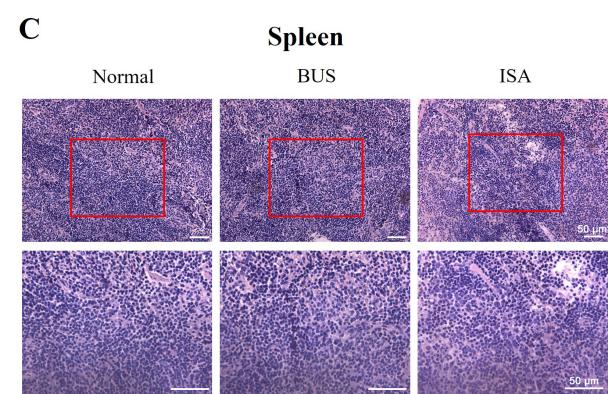

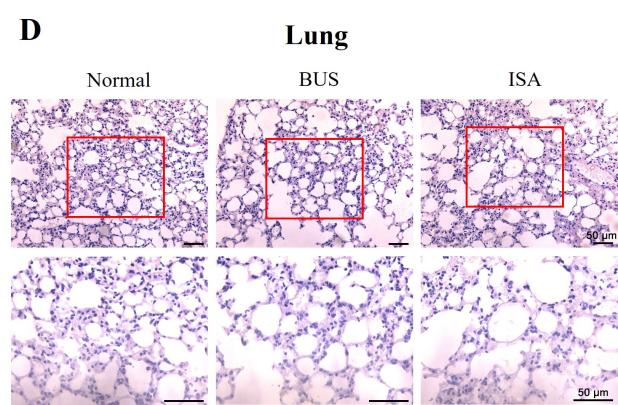

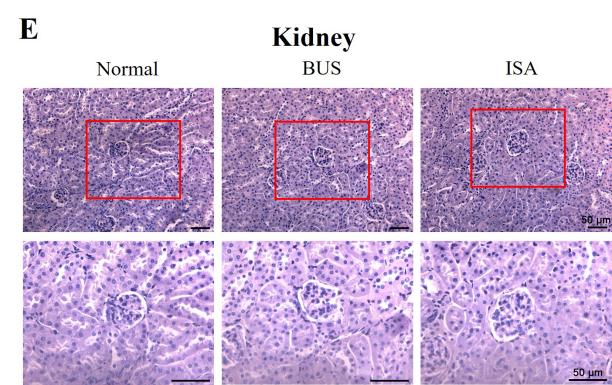


**Supplementary Figure 2.** Histological examination of mice organs. (A): Morphology of liver. (B): Morphology of heart. (C): Morphology of spleen. (D): Morphology of lung. (E): Morphology of kidney.

**Supplementary Table 1.** Primer Sequences for Real-Time Quantitative PCR.

| **Name** | **Primer Sequences** | **Tm** |
| --- | --- | --- |
| prm1-F | GCAGCAAAAGCAGGAGCAGATG | 70.21 |
| prm1-R | GGCAGCATCTTCGCCTCCTC | 70.57 |
| Tnp1-F | ATGGCATGAGGAGAGGCAAGAAC | 69.45 |
| Tnp1-R | TCGCCCCGTTTCCTACTTTTCAG | 70.62 |
| Tnp2-F | CTCGACACTCACCTGCAAGACC | 68.38 |
| Tnp2-R | GTCCGTTTCCGCCTCCTGAC | 70.23 |
| Tssk6-F | CGACAAACTCCTGAGCGAACTTG | 68.98 |
| Tssk6-R | GGCGGTCCACCACCTTGATG | 72.08 |
| H1f7-F | GCCAGAGCCAGGACACAAGAC | 68.43 |
| H1f7-R | GCTCCGATACGCACCTGTTCC | 69.9 |
| H2bc1-F | GGTGGCGGTAAAGGGTGCTAC | 68.42 |
| H2bc1-R | TCTCCTTGCGGCATCTCTTACG | 69.42 |
| H2al2a-F | GCAAAGGCGAAGAAGACGGAAAG | 70.57 |
| H2al2a-R | GCGAGGAATACAGGAGCAGAAGAG | 68.14 |
| Cabs1-F | AGAGGACAGTGCTGCTGATGTG | 66.85 |
| Cabs1-R | CTGAGGTGGCAGAGGAAGGAAC | 68.28 |
| Tppp2-F | ATGGATGGCAAGGCAGTGACC | 70.45 |
| Tppp2-R | GTTGATGGTTCTGGCATTCTTGGC | 71.23 |
| Prss37-F | TGCGTGGGTGTCCTCATCAAAG | 71.40 |
| Prss37-R | ATTGTCTGCTCTGTCCCATCTCTG | 67.65 |
| Pgk2-F | CATCGGGCTCACAGTTCTACGG | 69.79 |
| Pgk2-R | CCACCAAGGATAGCCAGGAAGG | 69.54 |
| Spem1-F | CCAACATCAACAACTGCCAGGAC | 69.61 |
| Spem1-R | TTCGGTGTAAGAGGATGCGGATTC | 70.65 |
| Prm3-F | TGAAGAAGCTCGTGGCCTGTG | 69.89 |
| Prm3-R | CCTCTTCCTCGTCCTCCTCCTC | 69.57 |
| Akap4-F | TCCGTGATGAAACTCTGCCTGATC | 70.36 |
| Akap4-R | CACATCTGGAGCCACTGCCTAC | 68.02 |
| Txndc2-F | CGTCCGAAGAAGACACCATCCAG | 70.86 |
| Txndc2-R | GATCACTCTCACCAAGCCTTCCTC | 68.24 |
| Garin2-F | GGCACCCGAAAACACACAGATAC | 68.33 |
| Garin2-R | TGATCCCGTTGTCCAGCATATCG | 71.72 |
| Gapdhs-F | AAGGCTGTAGGCAAAGTCATCCC | 68.15 |
| Gapdhs-R | CGGCAGGTCAGGTCCACAAC | 70.28 |
| Iqcf1-F | CAATGCTGGTGGCGGTTAATACTG | 69.73 |
| Iqcf1-R | GACTGCCCACTGCTCCTGTTG | 69.43 |
| Clxn-F | ATGGACCATGACCATGATGGGAAG | 71.60 |
| Clxn-R | TCTTTGGATCAGGCAGGCAAGG | 71.34 |
| Smcp-F | CGCTCAACCTACCTGCTGCTC | 68.32 |
| Smcp-R | CTGGTGGGGACTGTGGTGATTG | 70.83 |
